# KovaaK's aim trainer as a reliable metrics platform for assessing shooting proficiency in esports players: a pilot study

**DOI:** 10.3389/fspor.2024.1309991

**Published:** 2024-02-26

**Authors:** Ethan J. Rogers, Michael G. Trotter, Daniel Johnson, Ben Desbrow, Neil King

**Affiliations:** ^1^ Faculty of Health, School of Exercise and Nutrition Sciences, Queensland University of Technology, Brisbane, QLD, Australia; ^2^ Department of Psychology, Umeå University, Umeå, Sweden; ^3^ Faculty of Science, School of Computer Science, Queensland University of Technology, Brisbane, QLD, Australia; ^4^ School of Health Sciences and Social Work, Griffith University, Gold Coast, QLD, Australia; ^5^ Menzies Health Institute Queensland, Griffith University, Gold Coast, QLD, Australia

**Keywords:** esports, video game, esports performance, first-person shooter (FPS), performance, skill, reliability

## Abstract

Esports research lacks game-based metrics platforms appropriate for adequately capturing esports performance. The aim of this pilot study was to assess the reliability of the KovaaK's first-person shooter (FPS) aim trainer as a metrics platform for assessing shooting proficiency in esports players. Ten FPS esports players completed two identical experimental trials (T) separated by three to five days. Each trial included four rounds (R) of testing, evaluating four shooting tasks: Micro Flicking, Macro Flicking, Strafe Tracking, and Wall Peeking. Reliability of performance outcomes (e.g., accuracy, headshot accuracy, hits per second, and total shots hit) were assessed using the intraclass correlation coefficient (ICC) and their 95% confidence intervals (CI), and significant differences were identified using repeated-measures analysis of variance (RM-ANOVA). Results indicated excellent, or good to excellent reliability for all outcome variables with the ICC estimates ranging between 0.947–0.995, with lower and upper bound 95% CIs ranging between 0.876–0.988, and 0.984–0.999, respectively. Significant improvements were seen between experimental trials in the Macro Flicking task for accuracy (*p* = .005) and hits per second (*p* = .009) only. Significant interactions between trial and round were identified in the Micro Flicking task for accuracy (*p* = .006), with *post hoc* analysis showing accuracy was significantly higher in T1R1 compared to T2R1 (87.74 ± 3.13 vs. 85.99 ± 3.05, respectively, *p* = .02), and in T2R4 compared to T2R2 (87.99 ± 2.89 vs. 84.70 ± 4.25, respectively, *p* = .049). Significant interactions were also identified in the Strafe Tracking task for headshot accuracy (*p* = .002), with *post hoc* analysis showing headshot accuracy was significantly higher in T1R2 compared to T2R2 (78.48 ± 8.15 vs. 76.79 ± 12.16, respectively, *p* = .003), and in T1R2 compared to T1R1 (78.48 ± 8.15 vs. 73.68 ± 17.94, respectively, *p* = .023). In summary, this study demonstrates that KovaaK's provides a reliable metrics platform for assessing shooting proficiency in esports, however, some variability in performance was observed.

## 1 Introduction

Esports tournaments can be traced back to 1972, where players competed for a subscription to the Rolling Stones magazine (1). Recent professional esports tournaments offer prize pools of up to $40 million USD (2). In 2022, the esports industry had an estimated global audience of 532 million, and an estimated market value of $25 billion USD in 2019 (3, 4). Consensus on the definition of esports is lacking and presents challenges for research. However, a recent systematic review and thematic analysis on esports definitions has identified integral elements for classifying a video game as an esport (5). Thus, for the purpose of this paper, esports is defined as “*organised competitive digital gaming, played on a spectrum of professionalism*” (5). The authors acknowledge that other elements may be attributed to esports, such as tournaments and leagues or prize money, but are not considered necessary. Importantly, it should be noted that not all video games are esports.

Increasing financial incentives for professional esports athletes leads to the pursuit of heightened competition and performance optimization. Unlike other sports performance research, which has a well-established body of evidence with numerous validated performance metrics, esports performance research is underdeveloped and in its infancy. Understandably, the widely utilized traditional cognitive tests were not designed to capture the complex performance measures and characteristics associated with esports and therefore do not adequately capture the essence of esports performance. Additionally, in-game performance measures such as kill/death ratio, win/loss ratio and rank have been used to assess esports performance, yet inadequately capture individual skills due to extrinsic factors (e.g., teammates, opponents, etc.) (6). Hence, there is a need for more reliable, realistic, and objective performance metrics that can accurately evaluate individual skills and performance in esports (7). Once established, these outcomes could then be employed as standardized measures for assessing the performance-enhancing effects of interventions (e.g., dietary, psychological, etc.).

A challenge facing esports performance research is understanding the indicators of individual performance across the extensive ecosystem of games and genres. Various action performance indicators have been suggested to encapsulate individual performance in Counter-Strike: Global Offensive, and similar FPS esports, including reaction time, response time, keyboard proficiency, and mouse control (8). FPS video games rely on a combination of perceptual and motor skills (9), requiring efficient identification and processing of visual and auditory information to effectively execute coordinated movements using a mouse and keyboard, or controller (10). Shooting proficiency is a crucial aspect of mouse control in FPS games that demands effective visuomotor skills to eliminate enemies quickly and accurately. Shooting proficiency in esports players, often assessed via FPS aim training games, has been used to explore various modalities of research including the effects of caffeine interventions (11), and examining movement kinematics, motor acuity, and long-term motor learning (7, 12). FPS aim training games are used by players to enhance their visuomotor skills and shooting proficiency in other FPS games. Furthermore, aim training platforms offer players comprehensive performance data, encompassing performance measures such as shooting accuracy and hits/eliminations per second, making them potentially ideal performance metrics in esports research.

Prior to employing novel assessment tools in research, establishing their reliability is crucial to ensure the avoidance of type I and II errors (13). To our knowledge, two studies utilizing FPS aim trainers have included reliability assessments (11, 12). The 3D Aim Trainer (14) demonstrated good reliability for hit accuracy (ICC: 0.89) and excellent reliability for hit reaction time (ICC: 0.96) (11), while Aim Lab (15) demonstrated good to excellent reliability for hits per second (ICC: 0.88–0.96) and moderate to good reliability for hit accuracy on the Gridshot shooting task (ICC: 0.62–0.86) (12). The intraclass correlation coefficient (ICC) assesses the correlation and agreement between measures and is a suitable measure of reliability. ICC values less than 0.5 can be interpreted as “poor”, between 0.5 and 0.75 as “moderate”, between 0.75 and 0.9 as “good”, and greater than 0.9 as “excellent” (16). However, it is essential to understand that ICC estimates represent only the expected value of the true ICC, and should be interpreted with caution (13). A more appropriate approach is to consider the 95% confidence intervals (CI) of the ICC estimates, as they indicate where the true ICC estimate may fall (13). Therefore, in evaluating the reliability of previously employed FPS aim trainers, it is vital to make appropriate interpretations. As such, reliability of the 3D Aim Trainer is considered moderate to excellent for hit accuracy (95% CI [0.70, 0.97]), and good to excellent for hit reaction time (95% CI [0.88, 0.99]) (11). An appropriate interpretation of the reliability of Aim Lab is difficult, as the 95% CIs of the ICC estimates are not reported (12). Although the true ICC estimates may indeed infer excellent reliability of the 3D Aim Trainer and Aim Lab, the reported CIs, or lack thereof, suggest caution when drawing inferences from the measurements obtained.

Reliability assessments of FPS aim trainers yield valuable insights into the consistency of their measures to assess individual performance. Thus, it is questioned whether other widely used FPS aim trainers, such as KovaaK's (17), could provide more reliable measures. KovaaK's, unlike other FPS aim trainers, is developed in the Unreal Engine, offering the lowest input lag and perhaps more consistent measures. To our knowledge, KovaaK's has not been used in prior investigations. Thus, the aim of this investigation was to assess the reliability of KovaaK's to determine its suitability as a metrics platform for assessing shooting proficiency in esports.

## 2 Methods

### 2.1 Study design

A repeated measures reliability study was performed using a sample (*n* = 10) of FPS esports players. Each participant completed three sessions; one familiarization session followed by two identical experimental trials. All three sessions were three to five days apart. Experimental trials consisted of four rounds of testing involving four shooting tasks on KovaaK's. All sessions were conducted at the Games Research Laboratory at the Queensland University of Technology (QUT), Gardens Point, Brisbane, Australia. The study was reviewed and approved by the University Human Research Ethics Committee, in compliance with the National Statement on Ethical Conduct in Human Research (approval No. 5746). The participants provided their written informed consent to participate in this study.

### 2.2 Participants

Ten volunteer participants completed this study and were recruited via convenience and snowball sampling, including posts on relevant social media (e.g., Discord, Twitter, and Facebook), advertisement on the QUT research recruitment page, and posting flyers at local gaming cafes. Inclusion criteria for this study were ≥18 years of age and play ranked mode in an FPS game using mouse and keyboard controls. This study did not employ any exclusion criteria. The sample size was chosen due to the exploratory nature of the pilot study.

### 2.3 Pre-experimental procedures

Eligible participants completed an initial familiarization session where they practiced each of the four KovaaK's shooting tasks ten times each. This equated to 10 min of practice dedicated to each task, a duration chosen to provide a balance between providing sufficient practice without overburdening the participants. Previous research using similar FPS aim trainers have utilized a 6-min practice duration (7, 11). Throughout familiarization, participants were encouraged to adjust their mouse sensitivity to their preferred setting to be used in the subsequent experimental trials.

Standardized conditions within, and between participants between experimental trials were applied. Prior to the initial experimental trial, participants were asked to keep a record of their physical activity, sleep, and diet for 24 h, and repeat for the second experimental trial to standardize habitual patterns. These data served as a reference to check standardization for participants and were not analyzed in this study. Participants were also asked to refrain from playing the KovaaK's shooting tasks used in this study between trials with compliance checked verbally.

### 2.4 Experimental trial procedure

**Figure 1:**
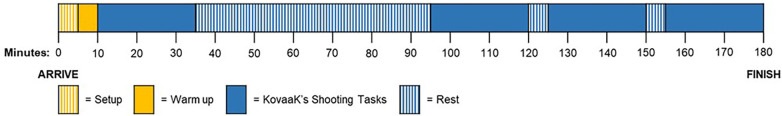
Testing procedure of the experimental trials.

The procedure for the experimental trials is shown schematically in Figure 1. Participants arrived at the laboratory at a time scheduled for their convenience (between 9 AM and 2 PM). On arrival, participants were asked to get comfortable at the computer where KovaaK's was pre-loaded with their preferred game settings from the familiarization session. Before testing commenced, participants completed one attempt at each KovaaK's shooting task as a warm-up. Immediately following the warm-up, participants began the first of four rounds of testing, each lasting approximately 25 min. Within each round, participants completed five attempts at each of the four shooting tasks, with rest periods between rounds. The baseline round was followed by a 60-min rest interval, with the subsequent three rounds separated by 5-min rest intervals. The 60-min rest interval was intended to assess the impact of a brief rest break in practice on subsequent performance for future intervention studies. During all rest periods participants were not permitted to engage in any mentally fatiguing activities (e.g., playing video games, reading, etc.) or consume food and beverages (except water).

### 2.5 Assessment measures—KovaaK's shooting tasks

KovaaK's (17) is available to download via Steam (https://store.steampowered.com/). It is designed for players to practice and improve their mechanical aiming skills via thousands of available shooting tasks. Generally, the tasks focus on either eliminating as many targets as possible, or accurately tracking targets for as long as possible. KovaaK's offers the ability to create novel shooting tasks or modify existing ones. For the purposes of this study, four existing shooting tasks were slightly modified. They were saved as Micro Flicking, Macro Flicking, Strafe Tracking, and Wall Peeking. The tasks can be found by clicking on the Online Scenario tab from the KovaaK's main menu and searching these terms via the search bar at the bottom of the screen. Each task was included due to their distinct motor movement requirements and relevance to different gameplay mechanics such as small flicks, large flicks, tracking, and a combination of player movements and flicks. “Flicking” is a skill in FPS games involving precise mouse movements to quickly and accurately eliminate enemies (18). Descriptions of each shooting task are explained below.

#### 2.5.1 Micro Flicking

**Figure 2:**
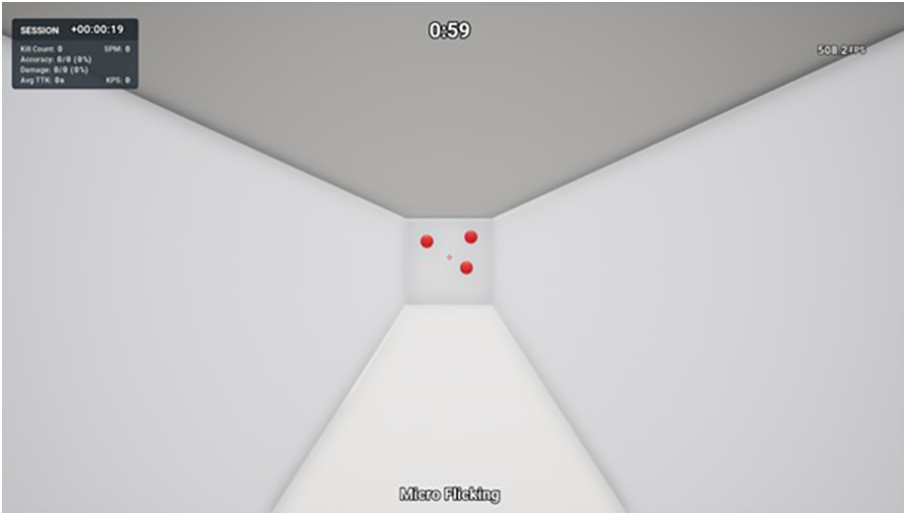
Micro Flicking—KovaaK's shooting task.

Micro Flicking can be considered a clicking task that involves clicking on static targets. The player spawns in a narrow lane with three small spherical targets present on a wall at the end (Figure 2). The goal is to eliminate the targets as quickly and accurately as possible for the entire 60 s duration. Following a successful elimination, a new target will spawn in a random location on the wall. Hence, there are three targets simultaneously present at any time. Players eliminate targets by moving the mouse to aim the cross crosshair over the target and clicking the left mouse button to shoot. Players can decide the sequence in which they eliminate the targets, as they have an unlimited duration. Performance outcome variables included for analysis in this study were accuracy (shots hit ÷ shots fired × 100) and number of shots hit per second (shots hit ÷ 60 s). This task involved small flicks, necessitated by the presence of smaller and closely spaced targets. Proficiency in small flicks is an essential skill in FPS games, enabling players to acquire targets quickly and accurately in close proximity to their initial crosshair placement. These small flicks may be employed either independently or immediately following large flicks, depending on the target's location on the screen.

#### 2.5.2 Macro Flicking

**Figure 3:**
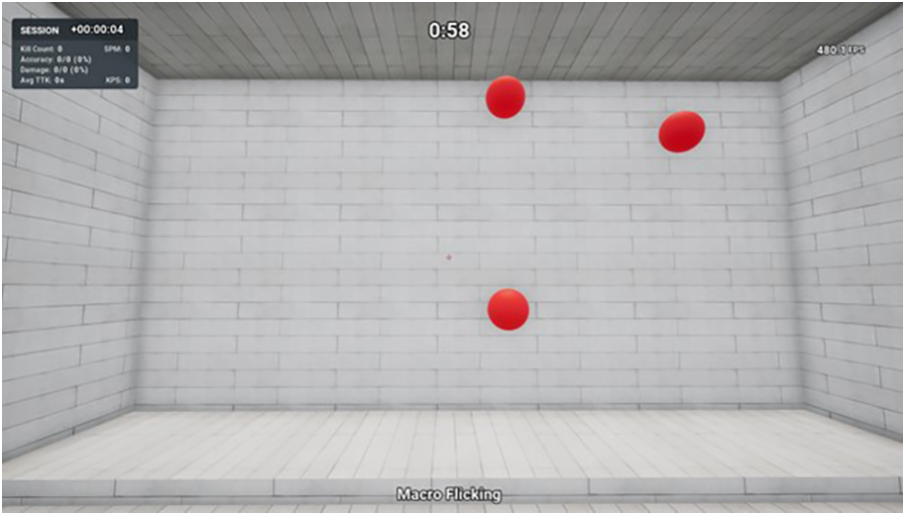
Macro Flicking—KovaaK's shooting task.

Macro Flicking can also be considered a clicking task, except the player spawns in a large room with three large spherical targets present on a wall in front of them (Figure 3). The goal is to eliminate the targets as quickly and accurately as possible for the entire 60 s duration. Following a successful elimination, a new target will spawn in a random location on the wall. Hence, there are three targets simultaneously present at any time. Players eliminate targets by moving the mouse to aim the cross crosshair over the target and clicking the left mouse button to shoot. Players can decide the sequence in which they eliminate the targets, as they have an unlimited duration. Performance outcome variables included for analysis in this study were accuracy (shots hit ÷ shots fired × 100) and number of shots hit per second (shots hit ÷ 60 s). This task involved executing large flicks, necessitated by the presence of larger and more distantly spaced targets. Proficiency in large flicks is an essential skill in FPS games, enabling players to rapidly reduce large distances between the initial crosshair placement and the target. Often, these large flicks are followed by a small flick to accurately acquire the target if the initial large flick was not sufficiently accurate.

#### 2.5.3 Strafe Tracking

**Figure 4:**
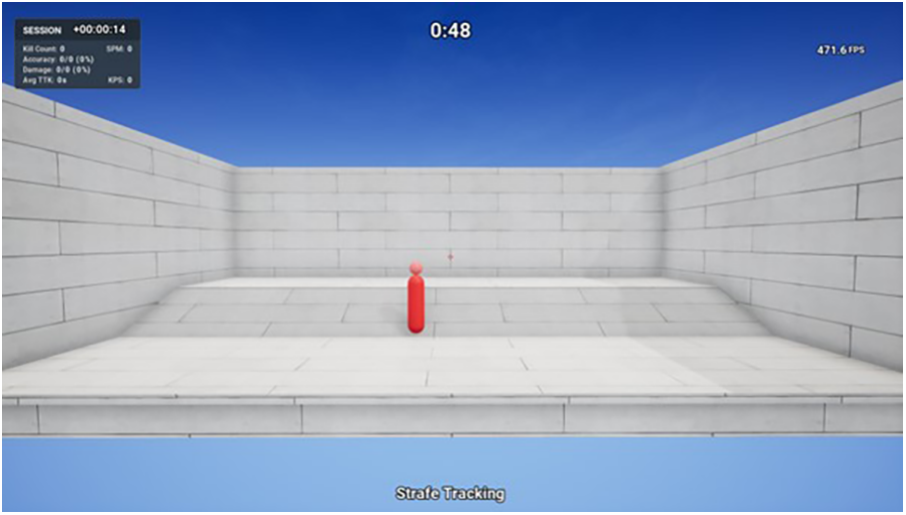
Strafe Tracking—KovaaK's shooting task.

Strafe Tracking can be considered a tracking task that involves tracking a moving target around the screen. The player spawns on a platform across from an open room with a bot in the middle that is comprised of a body (large pill shape) and head (small sphere shape) (Figure 4). The goal is to track the bot as accurately as possible for the entire 60 s duration, while it randomly moves around the room. The bot movements include forwards and backwards, side to side, and jumping. Players track the target by clicking and holding down the left mouse button to shoot while moving the mouse, attempting to always maintain the crosshair over the target. Headshots are worth two damage points and body shots are worth one damage point, hence, players may decide to prioritize headshots for a better score. In this study, participants were not instructed on how to strategize. Performance outcome variables included for analysis in this study were accuracy (shots hit ÷ shots fired × 100) and headshot accuracy (headshots hit ÷ total shots hit × 100). Tracking is an essential skill in FPS games, particularly in titles where enemies have a higher time to kill such as in Apex Legends. This necessitates the need for a greater number of accurate shots for a successful elimination. Conversely, in FPS games with a low time to kill such as Valorant, where a target can be eliminated with a single accurate shot, the emphasis shifts more towards precise flicks. Furthermore, the significance of tracking can be influenced by the fire rate of the chosen weapon. High fire rate weapons that deal less damage per bullet rely on tracking skill to deliver sufficient damage, opposed to single shot weapons with higher individual bullet damage, where reliance on flicks becomes more prominent.

#### 2.5.4 Wall Peeking

**Figure 5:**
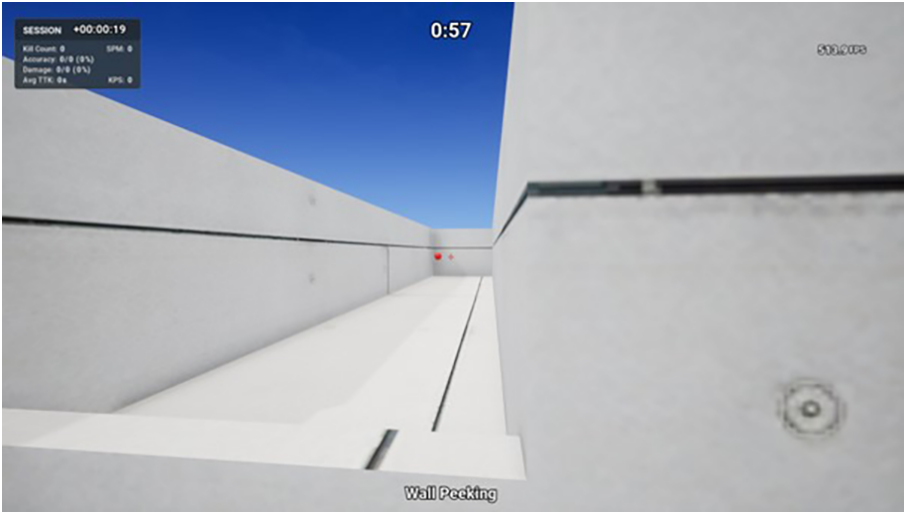
Wall Peeking—KovaaK's shooting task.

Wall Peeking can be considered a clicking task that involves clicking on static targets, except this time there is also character movement involved via the use of the keyboard. The player spawns facing a wall with two lanes either side of the wall and one spherical target spawns initially down the left lane (Figure 5). When eliminated, the next target will spawn in a random location down the right lane. When each target is eliminated, the next target will always spawn in a random location down the opposite lane, alternating left and right. The goal is to eliminate the targets as quickly and accurately as possible for the entire 60 s duration. Players eliminate targets by first using the keyboard to move their character to the left and when they visualize the target, move the mouse to aim the cross crosshair over the target and clicking the left mouse button to shoot. Using the keyboard, participants then moved their character to the right to eliminate the target down the right lane in the same manner, before moving back to the left lane. This process repeats for the entire 60 s duration. Movement keys are “w” to move forward, “s” to move backward, “a” to move left, and “d” to move right. Performance outcome variables included for analysis in this study were accuracy (shots hit ÷ shots fired × 100) and number of shots hit. This task involved a combination of character movement from behind cover and small flicks, resembling a crucial skill in various FPS games such as Valorant and CS:GO. In these titles, players strategically move from behind cover to quickly eliminate enemies that may appear, enabling players to efficiently navigate through the battlefield.

### 2.6 Statistical analysis

Statistical analysis was performed using IBM SPSS Statistics (Version 29). Normality was tested using the Shapiro-Wilk test. Data were log transformed and re-analyzed if non-normally distributed (Shapiro–Wilk test, *p* < .05). Two-way repeated measures analysis of variances (RM-ANOVAs) were performed to evaluate the effects of trial and round on each variable. Statistical significance was accepted as *p* < .05 and significant differences were identified with *post hoc* tests adjusted for multiple comparisons (Bonferroni). Data are reported as means ± standard deviations (SD). Reliability analysis was performed using the intraclass correlation coefficient (ICC). ICC estimates and their 95% confidence intervals (CI) were calculated based on a mean-rating (*k* = 8), absolute agreement, two-way mixed-effects model (13).

### 2.7 Data management

For each player, the average score from each round was computed for all performance outcome variables and used in subsequent analyses (i.e., the average of five attempts), yielding eight data points for each variable (one per round, with four rounds per trial, and two trials). This approach ensures a more accurate depiction of participants' true skill level, while mitigating the known influence of sub-optimal effort on performance (19).

## 3 Results

### 3.1 Participant characteristics

Ten participants (9 male, 1 female; age = 20.3 ± 1.8 years; weight = 76.7 ± 17.9 kg; BMI = 22.9 ± 4.5 kg/m^2^) completed the study. Two participants were current FPS professional esports athletes, three had previously played professionally, and the remaining five played recreationally. Participants self-reported an average of 29.1 ± 9.6 h per week playing video games, with 22.2 ± 7.9 h per week spent playing FPS games. Five participants played Valorant ranked in Platinum (*n* = 1), Diamond (*n* = 2), and Ascendant (*n* = 2). Four participants played Overwatch with individual ranks of #4402, #4300, #3596, and one top 500. One participant played Apex Legends ranked in Masters. Most participants (*n* = 8) reported previous use of FPS aim trainers, with half (*n* = 5) having previously used KovaaK's.

### 3.2 Reliability testing

All ICC estimates and their 95% CIs are shown in Table 1. The ICC estimates ranged from 0.947 to 0.995, with lower and upper bound 95% CI values ranging from 0.876 to 0.988, and from 0.984 to 0.999, respectively. Accuracy for Micro Flicking and Wall Peeking showed good to excellent reliability with their 95% CIs ranging between 0.876 and 0.985. All other variables demonstrated excellent reliability with 95% CIs >0.90.

**Table 1:** Reliability of all performance outcome variables from the KovaaK's shooting tasks, as measured by the intraclass correlation coefficient.

	ICC	95% CI	
		Lower	Upper
Micro Flicking			
Accuracy	.949	.882	.985
Hits per second	.989	.973	.997
Macro Flicking			
Accuracy	.958	.903	.988
Hits per second	.995	.988	.999
Strafe Tracking			
Accuracy	.988	.973	.997
Headshot accuracy	.972	.935	.992
Wall Peeking			
Accuracy	.947	.876	.984
Total shots hit	.965	.918	.990

<0.5 = poor; 0.5–0.75 = moderate; 0.75–0.9 = good; >0.9 = excellent; CI, confidence interval; ICC, intraclass correlation coefficient.

### 3.3 Effect of task repetition on shooting performance

All performance outcome variables are presented in Table 2. All assumptions of RM-ANOVAs were met unless otherwise specified.

**Table 2:** Results (means ± SDs) of all performance outcome variables from the KovaaK's shooting tasks.

	Micro Flicking		Macro Flicking		Strafe Tracking		Wall Peeking	
	ACC^a^	HPS^a^	ACC	HPS	ACC	HSA	ACC	TSH
T1	86.52 ± 3.40	3.66 ± 0.44	85.29 ± 3.38^d^	2.57 ± 0.34^d^	49.05 ± 8.28	76.39 ± 10.52	80.06 ± 7.85	43.10 ± 2.26
R1	87.74 ± 3.13^b^	3.60 ± 0.38	85.57 ± 3.43	2.54 ± 0.36	49.12 ± 8.13	73.68 ± 17.94^f^	80.27 ± 6.311	42.80 ± 1.96
R2	86.32 ± 3.16	3.62 ± 0.39	85.12 ± 3.67	2.58 ± 0.33	49.73 ± 7.77	78.48 ± 8.151^e,f^	79.72 ± 8.96	43.30 ± 2.20
R3	86.06 ± 4.14	3.68 ± 0.50	86.02 ± 4.28	2.58 ± 0.35	48.27 ± 9.36	77.59 ± 6.93	80.62 ± 9.08	43.06 ± 2.45
R4	85.95 ± 3.91	3.72 ± 0.48	84.47 ± 3.38	2.59 ± 0.33	49.07 ± 8.96	75.82 ± 11.61	79.62 ± 9.58	43.24 ± 3.26
T2	86.30 ± 2.99	3.70 ± 0.35	87.04 ± 3.74^d^	2.63 ± 0.31^d^	49.98 ± 7.72	77.24 ± 10.51	81.54 ± 6.70	44.43 ± 4.09
R1	85.99 ± 3.05^b^	3.62 ± 0.30	87.32 ± 3.39	2.59 ± 0.28	49.04 ± 7.84	75.41 ± 13.74	79.53 ± 9.69	43.72 ± 2.82
R2	84.70 ± 4.25^c^	3.67 ± 0.36	85.89 ± 4.87	2.63 ± 0.35	50.56 ± 8.81	76.79 ± 12.16^e^	82.00 ± 6.35	44.62 ± 4.02
R3	86.51 ± 3.29	3.71 ± 0.39	87.78 ± 4.46	2.65 ± 0.32	49.72 ± 7.86	77.78 ± 9.20	80.80 ± 7.81	44.32 ± 4.85
R4	87.99 ± 2.89^c^	3.81 ± 0.39	87.16 ± 3.97	2.65 ± 0.32	50.61 ± 6.96	78.96 ± 7.41	83.82 ± 7.59	45.04 ± 4.86

ACC, accuracy; HPS, hits per second; HSA, headshot accuracy; R, round; TSH, total shots hit; T, trial.

^a^ Indicates a significant main effect of round.

^b,c,e,f^ Indicates a significant trial × round interaction between pairs.

^d^ Indicates a significant main effect of trial.

#### 3.3.1 Micro Flicking—accuracy

There was no significant main effect of trial, *F*(1, 9) = 0.18, *p* > .05. There was a significant main effect of round, *F*(3, 27) = 3.761, *p* = .022, and there was a significant interaction between trial and round on accuracy, *F*(3, 27) = 5.17, *p* = .006, partial *η*^2^ = .365. Post hoc analysis showed that accuracy was significantly higher in Trial 1, Round 1 compared to Trial 2, Round 1 (T1R1 = 87.74 ± 3.13 vs. T2R1 = 85.99 ± 3.05, *p* = .02). Accuracy was also significantly higher in Trial 2, Round 4 compared to Trial 2, Round 2 (T2R4 = 87.99 ± 2.89 vs. T2R2 = 84.70 ± 4.25, *p* = .049). No other pairwise comparison differences were identified.

#### 3.3.2 Micro Flicking—hits per second

There was no significant main effect of trial, *F*(1, 9) = 1.27, *p* > .05. There was a significant main effect of round, *F*(1.379, 12.413) = 8.445, *p* < .001. Post hoc analysis showed hits per second was significantly higher in Round 4 compared to Round 1 (R4 = 3.77 ± 0.43 vs. R1 = 3.61 ± 0.34, *p* = .035), Round 2 (R4 = 3.77 ± 0.43 vs. R2 3.65 ± 0.37, *p* = .027), and Round 3 (R4 = 3.77 ± 0.43 vs. R3 = 3.70 ± 0.44, *p* = .005). There was no significant interaction between trial and round on hits per second, *F*(3, 27) = 1.904, *p* > .05.

#### 3.3.3 Macro Flicking—accuracy

There was a significant main effect of trial. Trial 2 was significantly higher than Trial 1 for accuracy (T2 = 87.04 ± 3.74 vs. T1 = 85.29 ± 3.38, *F*(1, 9) = 13.925, *p* = .005, partial *η*^2^ = .607). There was no significant main effect of round, *F*(3, 27) = 1.803, *p* > .05. There was no significant interaction between trial and round on accuracy, *F*(3, 27) = 0.839, *p* > .05.

#### 3.3.4 Macro Flicking—hits per second

There was a significant main effect of trial. Trial 2 was significantly higher than Trial 1 for hits per second (T2 = 2.63 ± 0.31 vs. T1 = 2.57 ± 0.34, *F*(1, 9) = 10.904, *p* = .009, partial *η*^2^ = .548). There was no significant main effect of round, *F*(3, 27) = 2.844, *p* > .05. There was no significant interaction between trial and round on hits per second, *F*(3, 27) = 0.167, *p* > .05.

#### 3.3.5 Strafe Tracking—accuracy

The assumption of sphericity was violated for round, thus significance was identified using the Greenhouse-Geisser correction. There was no significant main effect of trial, *F*(1, 9) = 1.836, *p* > .05. There was no significant main effect of round, *F*(1.391, 12.516) = 1.275, *p* > .05. There was no significant interaction between trial and round on accuracy, *F*(3, 27) = 0.516, *p* > .05.

#### 3.3.6 Strafe Tracking—headshot accuracy

There was no significant main effect of trial, *F*(1, 9) = 0.660, *p* > .05. There was no significant main effect of round, *F*(3, 9) = 1.000, *p* > .05. There was a significant interaction between trial and round on headshot accuracy, *F*(3, 27) = 6.530, *p* = .002, partial *η*^2^ = .420. Post hoc analysis showed that headshot accuracy was significantly higher in Trial 1, Round 2 compared to Trial 2, Round 2 (T1R2 = 78.48 ± 8.15 vs. T2R2 = 76.79 ± 12.16, *p* = .003). Headshot accuracy was also significantly higher in Trial 1, Round 2 compared to Trial 1, Round 1 (T1R2 = 78.48 ± 8.15 vs. T1R1 = 73.68 ± 17.94, *p* = .023).

#### 3.3.7 Wall Peeking—accuracy

There was no significant main effect of trial, *F*(1, 9) = 1.587, *p* > .05. There was no significant main effect of round, *F*(3, 27) = 0.758, *p* > .05. There was no significant interaction between trial and round on accuracy, *F*(3, 27) = 0.946, *p* > .05.

#### 3.3.8 Wall Peeking—total shots Hit

There was no significant main effect of trial, *F*(1, 9) = 0.091, *p* > .05. There was no significant main effect of round, *F*(3, 27) = 1.448, *p* > .05. There was no significant interaction between trial and round on accuracy, *F*(3, 27) = 0.610, *p* > .05.

## 4 Discussion

This study assessed the reliability of KovaaK's to determine its suitability as a metrics platform for assessing shooting proficiency in esports players. Reliability testing is essential when employing new assessment tools in research and is commonly assessed using the ICC to evaluate the degree of correlation and agreement between measurements (13). The main findings of this investigation were that KovaaK's demonstrated excellent reliability for six of the eight performance outcome variables analyzed across the four shooting tasks, while the remaining two demonstrated good to excellent reliability. Additionally, although most outcome variables showed no statistically significant differences over time, there was some variability.

To our knowledge, this study represents the first empirical utilization of KovaaK's. Hence, comparisons with previous findings remains a challenge and need to be made with alternative platforms [3D Aim Trainer (11); Aim Lab (12)]. Results of the present study indicate that KovaaK's may be a more reliable platform for assessing shooting proficiency in esports players. This conclusion is associated with the higher ICC estimates and their 95% CIs observed within this study for all outcome variables (11, 12). The disparity between KovaaK's and the 3D Aim Trainer, and Aim Lab, may indeed be attributed to the nuances of FPS aim trainers, however, it is essential to consider the methodological differences between studies. For instance, the present study included several rounds of testing within each trial, resulting in eight data points collected over two days. Each data point represents the average of five attempts, providing a more precise representation of a players skill level and reducing the within-subject variability. Comparatively, the reliability of the 3D Aim Trainer was assessed using an aggregate of three attempts between two trial days (11), while reliability of the Gridshot shooting task on Aim Lab was assessed on three to five individual attempts between different pairs of consecutive days (12). These methodological disparities may have contributed to the variations in reliability observed between aim trainers. Therefore, the higher ICC estimates and their 95% CIs observed in this study could be driven by both the KovaaK's platform and methodology employed. This warrants further exploration into methodological effects on reliability testing. Nonetheless, results of this study indicate a stronger level of agreement and correlation among the measurements obtained, enhancing the confidence in making inferences on the measurements obtained. This suggests that the results reliably reflect individual performance, with most of the observed variance attributed to between-subject variability rather than measurement error or within-subject variability. Therefore, KovaaK's can be used as a reliable tool for capturing individual shooting performance and be considered a reliable metrics platform for assessing shooting proficiency in esports players.

In addition to investigating the reliability of KovaaK's, this study also explored the effects of task repetition over time. The primary aim was to identify whether any statistically significant differences existed between trials for any of the performance outcome variables under question. There were significant improvements for accuracy and hits per second in Trial 2 compared to Trial 1 in the Macro Flicking task only. This finding is aligned with previous research using the Gridshot shooting task on Aim Lab, where significant improvements in accuracy and hits per second were also observed between days (12). In contrast, no differences in performance were seen between days on the 3D Aim Trainer (11), similar to the lack of statistically significant differences seen between trials for the other KovaaK's shooting tasks in this study. Direct comparisons with prior literature is difficult to make due to the distinct designs of the shooting tasks used in the respective studies. For instance, factors such as target size, distance between targets, target behavior, spawn locations, and environmental setting will vary between these tasks and may have an impact on the observed results. The significant improvements in the Macro Flicking task may be attributed to enhanced motor learning within this particular task, possibly due to the larger target size. Previous literature has shown that when players face smaller targets they strategically sacrifice speed for accuracy due to the higher demand on precision (distance to the center of the target) to be accurate, leading to a more strategic and perhaps consistent approach (7). As the Macro Flicking task has larger targets, participants may have strategically increased speed (hits per second) while simultaneously maintaining their level of accuracy, although shot precision may have decreased. Shot precision was not measured in this study and may warrant further exploration for a better understanding. Another potential explanation for the significant difference observed between trials could be performance retention, or practice effects, between trials. Participants performed similarly or better from the commencement of Trial 2 compared to the end of Trial 1. This was also observed for total shots hit in the Wall Peeking task, although no significant differences were observed in this task. Performance retention has previously been explored using the Gridshot shooting task on Aim Lab, where high levels of retention were seen for both accuracy and hits per second, which were highest among earlier days of practice (12). Thus, the Macro Flicking task may have been more facilitating of performance retention between trials and contributed to the statistically significant differences observed in accuracy and hits per second between trials. While Macro Flicking was the only task to observe statistically significant differences between trials, other changes in performance were observed (see Table 2). Although the differences were negligible and statistically insignificant, there was a trend for performance to be better in Trial 2 compared to Trial 1 across all performance outcome variables in all shooting tasks, except for accuracy in the Micro Flicking task. Notably, there is evidence of a practice effect occurring throughout the duration of the study, as all performance outcome variables showed the highest scores in the final round of the final experimental trial. Future investigations may wish to consider this practice effect and increase the number of familiarization sessions to gain more stability in participants performance before testing occurs.

Further to offering a suitable metrics platform for assessing shooting proficiency in esports research, these findings carry practical implications for professional esports athletes, teams and coaches seeking to evaluate the effects of different strategies on shooting performance. For instance, coaches wishing to investigate the impact of various factors such as ergogenic aids, sleep, equipment, game settings, and other variables on shooting performance can be confident that KovaaK's will provide an accurate representation of shooting proficiency when utilizing a similar methodological approach. Isolating shooting proficiency in a standardized environment such as KovaaK's provides valuable insight into this individual skill. However, it is important to acknowledge that caution is required if extrapolating to in-game environments. KovaaK's provides a controlled environment that optimizes cognitive focus on shooting targets, while the complex nature of FPS esports introduces various factors that increase cognitive load and potentially impact shooting performance during gameplay. Despite this uncertainty, the potential utility of KovaaK's lies in its capacity to act as a standardized platform for exploring how various factors, both in-game and external, influence shooting performance as a fundamental skill in all FPS games.

One key limitation was the relatively small sample size (*n* = 10). Additionally, although standardized pre-trial conditions within, and between participants between experimental trials were applied, it is acknowledged that there may have been some variability between participants, between experimental trials. Another limitation relates to the selection of the Strafe Tracking and Wall Peeking tasks. Firstly, the task design of Strafe Tracking may have introduced variability in scores that don't accurately represent performance. This was attributed to the nature of the target, comprising of a small sphere (representing the head) and larger pill shape (symbolizing the body), and participants discretion in aiming as they were not instructed to prioritize head or body shots in this task. Consequently, a participant's decision to prioritize one or the other could increase the variability in both outcome variables for this task. To mitigate this, future studies may wish to use an alternative tracking task featuring a single sphere target or give clear strategic directives to participants. Secondly, the Wall Peeking task had an intrinsic limiting factor of character movement. Between shots the necessary lateral character movement consumed a substantial portion of task duration reducing the actual time participants could spend shooting targets. Consequently, this task mechanic will have constrained performance and produced a lower task ceiling. For example, even under perfect performance with seamless and continuous lateral movement and shot accuracy, participants could only eliminate 60 targets within the 60 s task duration, assuming a one second traverse between sides. Thus, this task is not an appropriate choice for assessing solely shooting performance but may be considered with inclusion of combined keyboard/movement proficiency.

In conclusion, FPS aim trainers have proven to be suitable metrics platforms for assessing individual skill in FPS esports players. Despite some natural variability in performance across time, this study has successfully shown that KovaaK's is a reliable metrics platform for assessing shooting proficiency and could be used in esports research. However, there are important considerations for future research including an increased sample size, inclusion of more professional esports athletes, task choice, and potential natural performance differences over time that may be diminished via increased familiarization sessions. A practical recommendation for future studies is to use a similar methodological approach by averaging scores from multiple attempts across more time points, as this will help gain a better representation of an individual's true skill level.

## Data Availability

The raw data supporting the conclusions of this article will be made available by the authors, without undue reservation.
